# Gas–liquid mass transfer characteristics of aviation fuel scrubbing in an aircraft fuel tank

**DOI:** 10.1038/s41598-021-94786-1

**Published:** 2021-07-29

**Authors:** Li Chaoyue, Feng Shiyu, Xu Lei, Peng Xiaotian, Yan Yan

**Affiliations:** 1 School of Mechanical and Electrical Engineering, Jinling Institute of Technology, Nanjing, China; 2 Key Laboratory of Aircraft environment control and life support, MIIT, Nanjing University of Aeronautics and Astronautics, Nanjing, China; 3 College of Mechanical and Electrical Engineering, Xi’an Polytechnic University, Xi’an, China

**Keywords:** Energy science and technology, Engineering

## Abstract

Dissolved oxygen evolving from aviation fuel leads to an increase in the oxygen concentration in an inert aircraft fuel tank ullage that may increase the flammability of the tank. Aviation fuel scrubbing with nitrogen-enriched air (NEA) can largely reduce the amount of dissolved oxygen and counteract the adverse effect of oxygen evolution. The gas–liquid mass transfer characteristics of aviation fuel scrubbing are investigated using the computational fluid dynamics method, which is verified experimentally. The effects of the NEA bubble diameter, NEA superficial velocity and fuel load on oxygen transfer between NEA and aviation fuel are discussed. Findings from this work indicate that the descent rate of the average dissolved oxygen concentration, gas holdup distribution and volumetric mass transfer coefficient increase with increasing NEA superficial velocity but decrease with increasing bubble diameter and fuel load. When the bubble diameter varies from 1 to 4 mm, the maximum change of descent rate of dissolved oxygen concentration is 18.46%, the gas holdup is 8.73%, the oxygen volumetric mass transfer coefficient is 81.45%. When the NEA superficial velocities varies from 0.04 to 0.10 m/s, the maximum change of descent rate of dissolved oxygen concentration is 146.77%, the gas holdup is 77.14%, the oxygen volumetric mass transfer coefficient is 175.38%. When the fuel load varies from 35 to 80%, the maximum change of descent rate of dissolved oxygen concentration is 21.15%, the gas holdup is 49.54%, the oxygen volumetric mass transfer coefficient is 44.57%. These results provide a better understanding of the gas and liquid mass transfer characteristics of aviation fuel scrubbing in aircraft fuel tanks and can promote the optimal design of fuel scrubbing inerting systems.

## Introduction

Fuel tank explosions are one of the main causes of aircraft crashes. Research has illustrated that fuel tanks burn easily when an external ignition source exists if the oxygen concentration in the ullage exceeds the limiting oxygen concentration (LOC)^1,2^. Considering the differences in ignition energy between military and civilian aircraft, the LOC is set as 12% for civilian aircraft and 9% for military aircraft^3,4^. Fuel tank inerting technology is practical and widely used for the protection of aircraft fuel tanks. NEA is injected into tank ullage to displace oxygen and reduce the oxygen concentration to less than the LOC^5,6^. However, dissolved oxygen may be released from aviation fuel due to the concentration difference between the inert ullage and fuel during flight. The released oxygen entering the ullage results in an increase in the ullage oxygen concentration and makes the fuel tank combustible^7^. To counteract the effect of dissolved oxygen evolution, fuel scrubbing inerting has been proposed to pump NEA into the fuel to displace the dissolved oxygen and has been applied to military aircraft^8,9^.

Fuel scrubbing is a simple and enhanced gas–liquid direct contact deoxygenation method. Dissolved oxygen evolves and enters an NEA bubble across the interface between gas and liquid because of the difference in oxygen partial pressure. The oxygen mass transfer performance is closely related to the hydrodynamics. Unfortunately, there is a lack of research on oxygen mass transfer in aviation fuel scrubbing. The gas–liquid mass transfer performance in aeration tanks and bubble columns is similar to fuel scrubbing, and there are numerous studies on the parameters affecting oxygen mass transfer^10,11^.

Gillot^12^ experimentally studied the bubble size and oxygen mass transfer in an oxidation ditch affected by horizontal flow, and the results indicated that horizontal velocity could enhance the oxygen transfer and bubble diameter. Kulkarni^13^ performed experiments to investigate the effects of bubble size distribution on mass transfer in a bubble column reactor. Buwa^14^ experimentally studied the effects of sparger design, gas superficial velocity and coalescence suppressing additives on gas–liquid flow dynamics. Trivedi^15^ conducted an experiment to study the hydrodynamics of countercurrent bubbles, and the results showed that bubble diameter decreases with increasing liquid velocity. McClure^16^ measured the oxygen transfer rate, bubble size, interfacial area and volumetric mass transfer coefficient in a bubble column, which are useful parameters for predicting the gas mass transfer characteristics in theoretical calculations.

In addition, the CFD method is also widely used in gas–liquid direct contact mass transfer. Terashima^17^ studied the effects of bubble size on the volumetric oxygen coefficient in different aeration tanks. Fayolle^18^ studied the axial liquid velocities, local gas holdups and oxygen transfer coefficients in four different aeration tanks based on the CFD method. Wen^19^ investigated the mass transfer coefficient between regeneration air and liquid desiccant in a liquid desiccant cooling system using CFD. Talvy^20^ simulated the hydrodynamics and axial dispersion of two-phase bubbly flow in an airlift internal loop reactor with CFD. Gresch^21^ found that CFD is a valuable and accurate tool for simulating the flow field in aeration tanks.

The oxygen mass transfer in fuel scrubbing directly affects the potential increase in ullage oxygen concentration. For the optimized design of a fuel scrubbing inerting system, the oxygen transfer characteristics affected by the NEA bubble size, NEA superficial velocity and fuel load are studied using an experimentally verified CFD model.

## Physical and mathematical models

### Physical model

**Figure 1:**
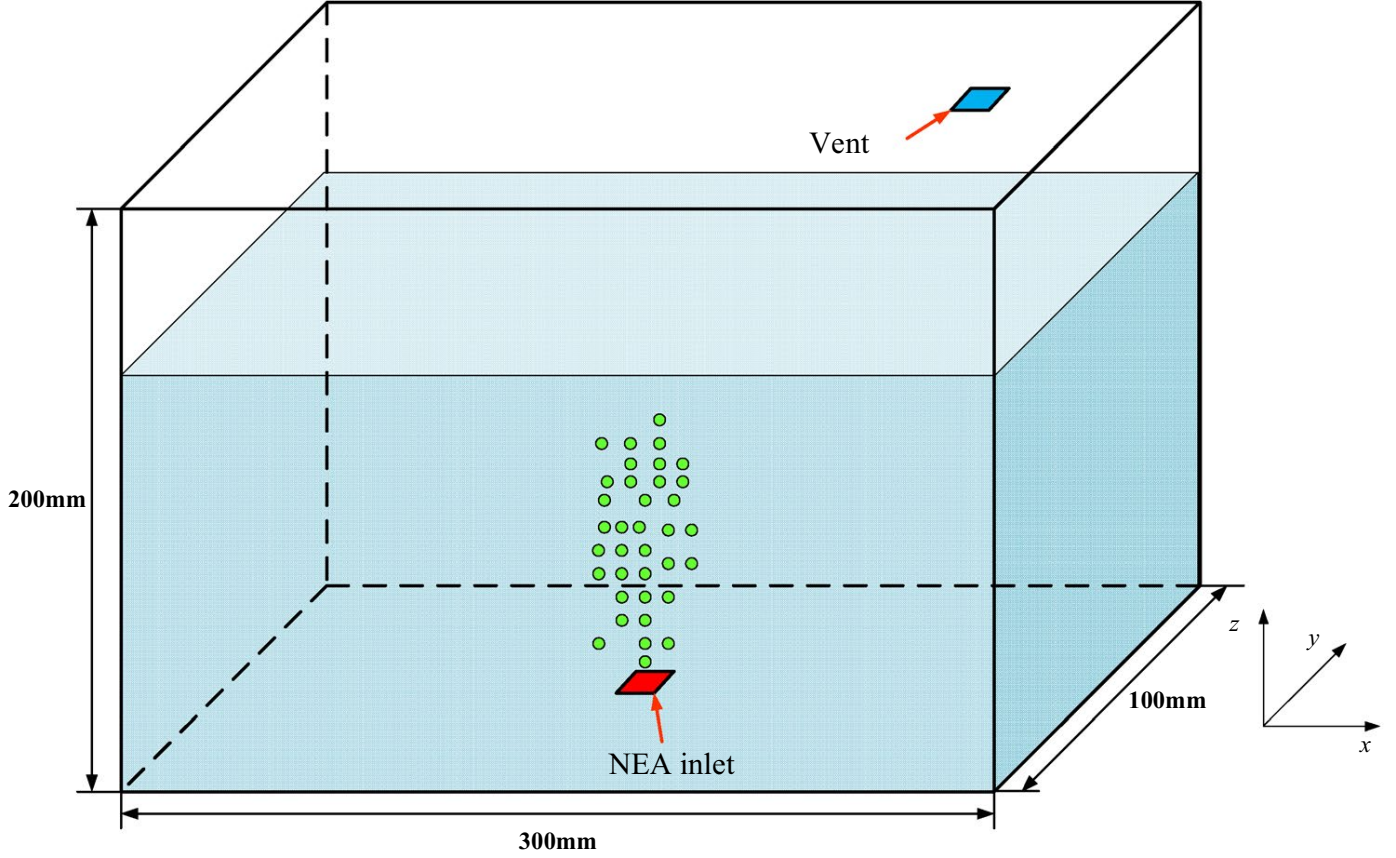
Schematic diagram of the scaled fuel tank.

A schematic diagram of the scaled fuel tank is sketched in Fig. 1, and the three-dimensional model size is 300 × 200 × 100 mm^3^. The tank is filled with aviation fuel to a predetermined level. Then, NEA is injected into the aviation fuel from an inlet sparger at the bottom of the fuel tank, forming tiny bubbles that are dispersed throughout the liquid.

### Mathematical model

#### Conservation equations

The Euler–Euler two-fluid model is suitable and widely used in CFD simulations of gas–liquid direct contact mass transfer^22,23^. In the Euler–Euler two fluid model, liquid as a continuous phase is interspersed with dispersed gas. Each phase in the multiphase flow model is solved by a set of momentum equations and continuity equation models. The conservation equations for the Euler–Euler model are shown below.

The mass conservation equation for the gas or liquid phase can be expressed as:

1 $$ \frac{\partial }{\partial t}(\rho_{{\text{i}}} \alpha_{{\text{i}}} ) + \nabla (\rho_{{\text{i}}} \alpha_{{\text{i}}} {\mathbf{u}}_{{\text{i}}} ) = 0 $$

The momentum conservation for both phases can be written as:

2 $$ \frac{\partial }{{\partial t}}(\rho _{{\text{i}}} \alpha _{{\text{i}}} {\mathbf{u}}_{{\text{i}}} ) + \nabla  \cdot (\rho _{{\text{i}}} \alpha _{{\text{i}}} {\mathbf{u}}_{i} {\mathbf{u}}_{i} ) =  - \alpha _{i} \nabla p + \nabla  \cdot \left[ {\alpha _{i} \mu _{{\text{i}}} (\nabla {\mathbf{u}}_{i}  + \nabla {\mathbf{u}}_{i} ^{{\text{T}}} )} \right] + \alpha _{i} \rho _{i} {\mathbf{g}} + {\mathbf{R}}_{{i{\text{j}}}}$$

**R**_ij_ is the interfacial force between the gas and liquid, which mainly includes the drag force, lift force and virtual mass force. Because the forces between the gas and liquid phases are reciprocal, the interfacial forces of each phase can be expressed as:

3 $$ {\mathbf{R}}_{{\text{l}}} = - {\mathbf{R}}_{{\text{g}}} = {\mathbf{R}}_{{\text{l}}}^{{{\text{DF}}}} + {\mathbf{R}}_{{\text{l}}}^{{{\text{LF}}}} + {\mathbf{R}}_{{\text{l}}}^{{{\text{VMF}}}} $$

The drag force is essentially the frictional resistance between the bubble and liquid during the gas–liquid flow, which can be expressed as:

4 $$ {\mathbf{R}}_{{\text{l}}}^{{{\text{DF}}}} = \frac{3}{4}\rho_{l} \alpha_{g} \frac{{C_{{\text{D}}} }}{{d_{{\text{B}}} }}({\mathbf{u}}_{g} - {\mathbf{u}}_{l} )|{\mathbf{u}}_{g} - {\mathbf{u}}_{l} | $$

where (**u**_g_–**u**_l_) is the slip velocity between the gas and liquid. *C*_D_ is determined by the bubble Reynold number and Eotvos number, which is presented as^14,24^:

5 $$ C_{{\text{D}}} = \max \left( {\min \left( {\frac{24}{{Re}}(1 + 0.15Re^{0.687} ),\frac{72}{{Re}}} \right),\frac{8}{3}\frac{Eo}{{Eo + 4}})} \right) $$

The bubble Reynold number and Eotvos number can be expressed as^25^:

6 $$ {\text{Re}} = \frac{{\rho_{{\text{l}}} d_{{\text{B}}} |{\mathbf{u}}_{l} - {\mathbf{u}}_{g} |}}{{\mu_{l} }} $$

7 $$ Eo = \frac{{|\rho_{{\text{g}}} - \rho_{{\text{l}}} |gd_{B}^{2} }}{8\omega } $$

The momentum exchange between bubbles and liquid in two-phase flow due to aerodynamic lift can be expressed in terms of lift force and can be expressed as^26^:

8 $$ {\mathbf{R}}_{{\text{l}}}^{{{\text{FL}}}} = \alpha_{{\text{g}}} \rho_{{\text{l}}} C_{{\text{L}}} ({\mathbf{u}}_{g} - {\mathbf{u}}_{l} ) \times \nabla \times {\mathbf{u}}_{l} $$

where *C*_L_ is the lift coefficient with a value of 0.5^27^.

The virtual mass force is the force exerted on the surrounding liquid by the accelerated motion of the bubble and can be expressed as:

9 $$ {\mathbf{R}}_{{\text{l}}}^{{{\text{VMF}}}} = C_{{{\text{VM}}}} \rho_{{\text{l}}} \alpha_{{\text{g}}} \left( {\frac{{{\text{d}}{\mathbf{u}}_{{\text{g}}} }}{{{\text{d}}t}} - \frac{{{\text{d}}{\mathbf{u}}_{{\text{l}}} }}{{{\text{d}}t}}} \right) $$

where *C*_VM_ is the virtual mass coefficient with a set value of 0.5 for accurate results^28^.

#### Turbulence model

The main function of the turbulence model is to solve the turbulent motion equation of the fluid by connecting the new unknowns with the average velocity gradient. Many turbulence models have been developed in the literature^29,30^, and the two equation models are the most straightforward turbulence models to obtain the velocity scale turbulent kinetic energy *k* and turbulence length scale dissipation rate *ε* by solving two additional transport Eqs. ^31^. The standard *k*–*ε* model is widely used in academia and industry because of its advantages in robustness, economics and accuracy in computation^32,33^. The standard *k*–*ε* model can be expressed as:

10 $$ \frac{\partial }{\partial t}(\alpha_{{\text{l}}} \rho_{{\text{l}}} k_{{\text{l}}} ) + \nabla (\alpha_{{\text{l}}} \rho_{{\text{l}}} {\mathbf{u}}_{{\text{l}}} k_{{\text{l}}} ) = \nabla \cdot (\alpha_{{\text{l}}} (\mu + \frac{{\mu_{{\text{t}}} }}{{\omega_{k} }})\nabla k_{{\text{l}}} ) + \alpha_{{\text{l}}} (P_{k} - \rho_{{\text{l}}} \varepsilon_{{\text{l}}} ) $$

11 $$ \frac{\partial }{\partial t}(\alpha_{{\text{l}}} \rho_{{\text{l}}} \varepsilon_{{\text{l}}} ) + \nabla (\alpha_{{\text{l}}} \rho_{{\text{l}}} {\mathbf{u}}_{{\text{l}}} \varepsilon_{{\text{l}}} ) = \nabla \cdot (\alpha_{{\text{l}}} (\mu + \frac{{\mu_{{\text{t}}} }}{{\omega_{\varepsilon } }})\nabla \varepsilon_{{\text{l}}} ) + \alpha_{{\text{l}}} \frac{{\varepsilon_{{\text{l}}} }}{{k_{{\text{l}}} }}(C_{1} P_{k} - C_{2} \rho_{{\text{l}}} \varepsilon_{l} ) $$

where *ω*_k_, *ω*_ε_, *C*_1_ and *C*_2_ can be set to values of 1.0, 1.3, 1.44 and 1.92, respectively.

The turbulent viscosity *μ*_t_ can be expressed as:

12 $$ \mu_{{\text{t}}} = \frac{{C_{{\upmu }} \rho_{{\text{l}}} k_{{\text{l}}}^{2} }}{{\varepsilon_{{\text{l}}} }} $$

where *C*μ is an empirical coefficient that can be set as 0.09.

#### Two-phase mass transfer

The quantity of oxygen mass transfer between the gas bubble and liquid is solved by the general transport equation in two-phase flow, which can be expressed as:

13 $$ \frac{{\partial \alpha _{{\text{i}}} c_{{\text{i}}} }}{{\partial t}} + \nabla  \cdot (\alpha _{{\text{i}}} c_{{\text{i}}} {\mathbf{u}}_{{\text{i}}} ) =  - \nabla  \cdot (\alpha _{{\text{i}}} {\text{(}}{\mathbf{J}}_{{\text{i}}} {\text{ + }}c^{\prime}_{{\text{i}}} {\text{u}}^{\prime}_{{\text{i}}} {\text{) + }}S_{{\text{i}}}$$

During scrubbing, the change in oxygen and nitrogen partial pressure results in oxygen and nitrogen mass transfer between the NEA bubble and aviation fuel. It can be expressed as:

14 $$ S_{{\text{O}}} = K_{{\text{O}}} a(c_{{\text{s,O}}} - c_{{\text{O}}} ) $$

15 $$ S_{{\text{N}}} = K_{{\text{N}}} a(c_{{\text{s,N}}} - c_{N} ) $$

The saturation concentration of O_2_ and N_2_ in aviation fuel can be determined according to the Ostwald coefficient, which represents the volume of gas dissolved per volume of liquid at the specified partial pressure of gas and temperature. The Ostwald coefficient^34^ can be expressed as:

16 $$ L = \frac{{2.31(980 - \rho_{{\text{l}}} )}}{1000}\exp \left[\frac{0.639(700 - T)}{T}ln(3.333L_{0} )\right] $$

where *L*_0_ is the Ostwald coefficient at 0 ℃ for petroleum liquids with *ρ*_l_ = 850 kg/m^3^, which are set to values of 0.16 and 0.069 for O_2_ and N_2_, respectively.

The gas–liquid interface area is determined by the ratio of the total bubble surface and the volume of liquid, and it can be expressed as:

17 $$ a = \frac{6}{{d_{{{\text{bs}}}} }}\frac{{\alpha_{{\text{g}}} }}{{1 - \alpha_{{\text{g}}} }} $$

where *d*_bs_ is the Sauter diameter of the NEA bubble and is given as^11^:

18 $$ d_{{{\text{bs}}}} = \frac{{\sum {n_{{\text{q}}} d_{{{\text{bq}}}}^{3} } }}{{\sum {n_{{\text{q}}} d_{{{\text{bq}}}}^{{2}} } }} $$

19 $$ d_{{{\text{bq}}}} = (h_{{{\text{bq}}}} l_{{{\text{bq}}}}^{2} )^{\frac{1}{3}} $$

The classic penetration theory^35^ is applied to determine the mass transfer coefficient and is expressed as:

20 $$ K_{{\text{O}}} = 2\sqrt {\frac{{D_{{\text{O}}} u_{{\text{r}}} }}{{\pi d_{{{\text{bs}}}} }}} $$

21 $$ K_{{\text{N}}} = 2\sqrt {\frac{{D_{{\text{N}}} u_{{\text{r}}} }}{{\pi d_{{{\text{bs}}}} }}} $$

The mass diffusion coefficient is closely related to viscosity, temperature and other factors^36,37^. In our previous studies, the authors experimentally measured the oxygen and nitrogen mass diffusion coefficients in RP3 aviation and correlated them as follows^38,39^:

22 $$ D_{{\text{O}}} = \frac{1}{\mu }(6.632 \times 10^{ - 16} T^{2} - 1.351 \times 10^{ - 13} T) $$

23 $$ D_{{\text{N}}} = \frac{1}{\mu }(1.806 \times 10^{ - 16} T^{2} - 2.087 \times 10^{ - 14} T) $$

## Numerical solution

### Simulation details

The gas–liquid mass transfer characteristics of aviation fuel scrubbing are studied by solving the equations above with commercial ANSYS-Fluent 18.0 software. The simulations are performed with the Euler-Euler two-fluid model. The mass transfer between gas and liquid is simulated by applying a define function to the loading mass transfer source term at the gas–liquid interface. The liquid RP3 aviation fuel (*ρ*_l_ = 805 kg/m^3^; *μ*_l_ = 1.15 × 10^–3^ Pa∙s) is a continuous phase, and NEA (N_2_ and O_2_ volume fractions are 95% and 5%, respectively) is a dispersed phase. The simulations are conducted at a constant temperature of 300 K.

**Figure 2:**
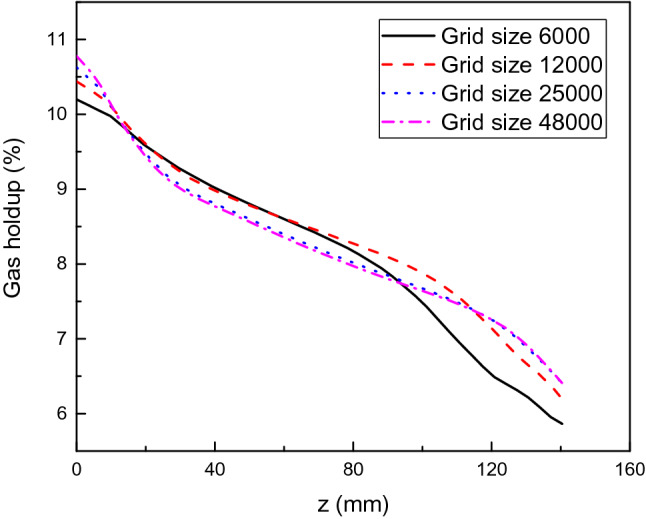
Gas holdup distributions with four types of grids.

To ensure the accuracy of the simulation, grid independence verification must be carried out in advance. Grid sizes of 6000, 12,000, 25,000 and 48,000 are created, and the scrubbing process is simulated with a fuel load of 80% and an NEA superficial velocity of 0.04 m/s. The gas holdup distributions on the straight line between the two coordinate points (150, 50, 0) and (150, 50, 160) in the tank are presented in Fig. 2. It is obvious that a grid size of 25,000 is sufficient for the simulation considering the calculation accuracy and cost.

### Simulation correctness verification

**Figure 3:**
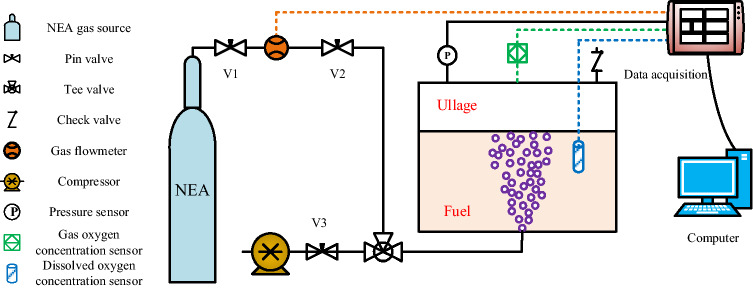
The experimental fuel scrubbing system device.

The variation in dissolved oxygen concentration during aviation fuel scrubbing directly reflects the gas–liquid mass transfer characteristics. Therefore, the dissolved oxygen concentration is calculated by the CFD method and compared with experiments to verify the correctness of the simulation. The experimental device system of fuel scrubbing is presented in Fig. 3. The dissolved oxygen concentration was monitored by a Figaro KDS-25B oxygen concentration sensor with a measurement range of 0 ~ 80 mg/L.

**Figure 4:**
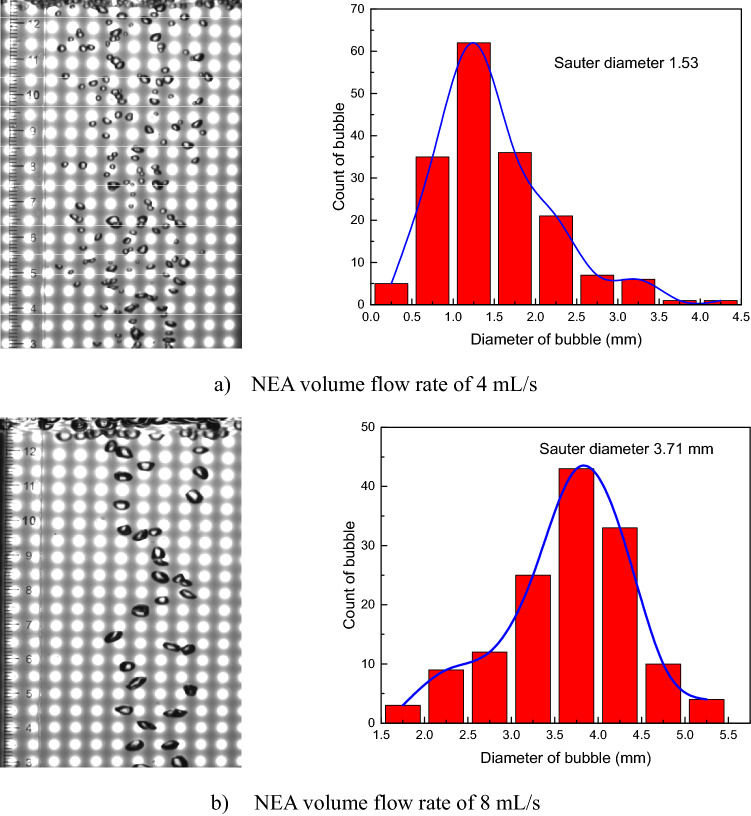
NEA bubble shape and equivalent diameter distribution.

The bubble diameter is essential for determining the mass transfer coefficients of O_2_ and N_2_ according to Eqs. (21) and (22). The NEA bubble shape in the fuel is photographed by a charge-coupled device (CCD), and the minor and major axes can be obtained with the help of the digital image processing software ImageJ^40^. Therefore, the Sauter diameters calculated by Eqs. (19) and (20) are used as an important input parameter in the CFD simulation. Two different experimental cases under NEA volume flow rates of 4 mL/s and 8 mL/s at a fuel load of 80% are conducted. The NEA bubble shape and equivalent diameter distribution in the fuel are presented in Fig. 4. The Sauter diameter was calculated to be 1.53 mm at an NEA volume flow rate of 4 mL/s and 3.71 mm at 8 mL/s.

**Figure 5:**
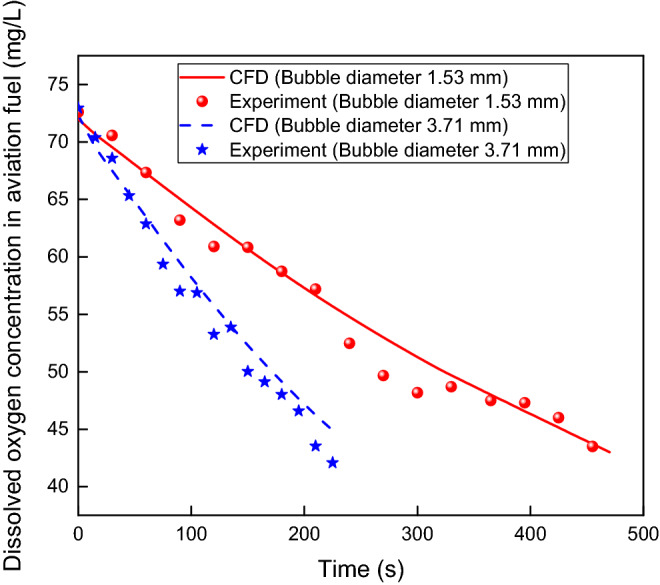
Comparison of dissolved oxygen concentrations between CFD simulation and experiment.

The CFD simulation and experiment were conducted under the conditions above, and the ullage oxygen concentration was decreased to 12%. The comparison of dissolved oxygen concentrations between the CFD simulation and experiment is shown in Fig. 5. The maximum deviation between the CFD and experiment is less than 6.25% for a Sauter diameter of 1.53 mm and 6.67% for a Sauter diameter of 3.71 mm. Despite a small deviation, the CFD simulations are in good agreement with the experiment, and the CFD method can be applied to accurately study the gas–liquid mass transfer characteristics of aviation fuel scrubbing.

## Results and discussion

In the aviation fuel scrubbing process, the characteristics of gas–liquid mass transfer vary with NEA bubble diameter, NEA superficial velocity and fuel load. The influence of these parameters is studied below.

### Influence of NEA bubble diameter

**Figure 6:**
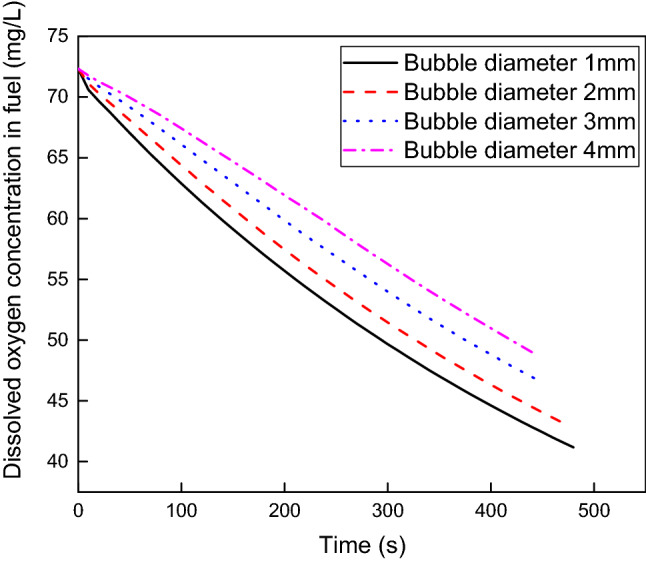
Dissolved oxygen concentration versus scrubbing time at various bubble diameters.

The gas-liquid mass transfer in aviation fuel scrubbing is simulated with different NEA bubble diameters of 1 mm, 2 mm, 3 mm and 4 mm at a superficial NEA velocity of 0.04 m/s and fuel load of 80%. Figure 6 presents the dissolved oxygen concentration versus scrubbing time. The dissolved oxygen concentration decreases with increasing scrubbing time, and the larger the bubble diameter is, the greater the dissolved oxygen concentration. The maximum difference in dissolved oxygen concentration at the same scrubbing time can be as great as 14.68% at these four bubble diameters. The rate of descent of dissolved oxygen concentration decreases as the bubble diameter increases. The average rates of descent of dissolved oxygen concentration are 0.065 mg/s, 0.062 mg/s, 0.057 mg/s and 0.053 mg/s when the bubble diameter varies from 1 to 4 mm, and the maximum change is 18.46%.

**Figure 7:**
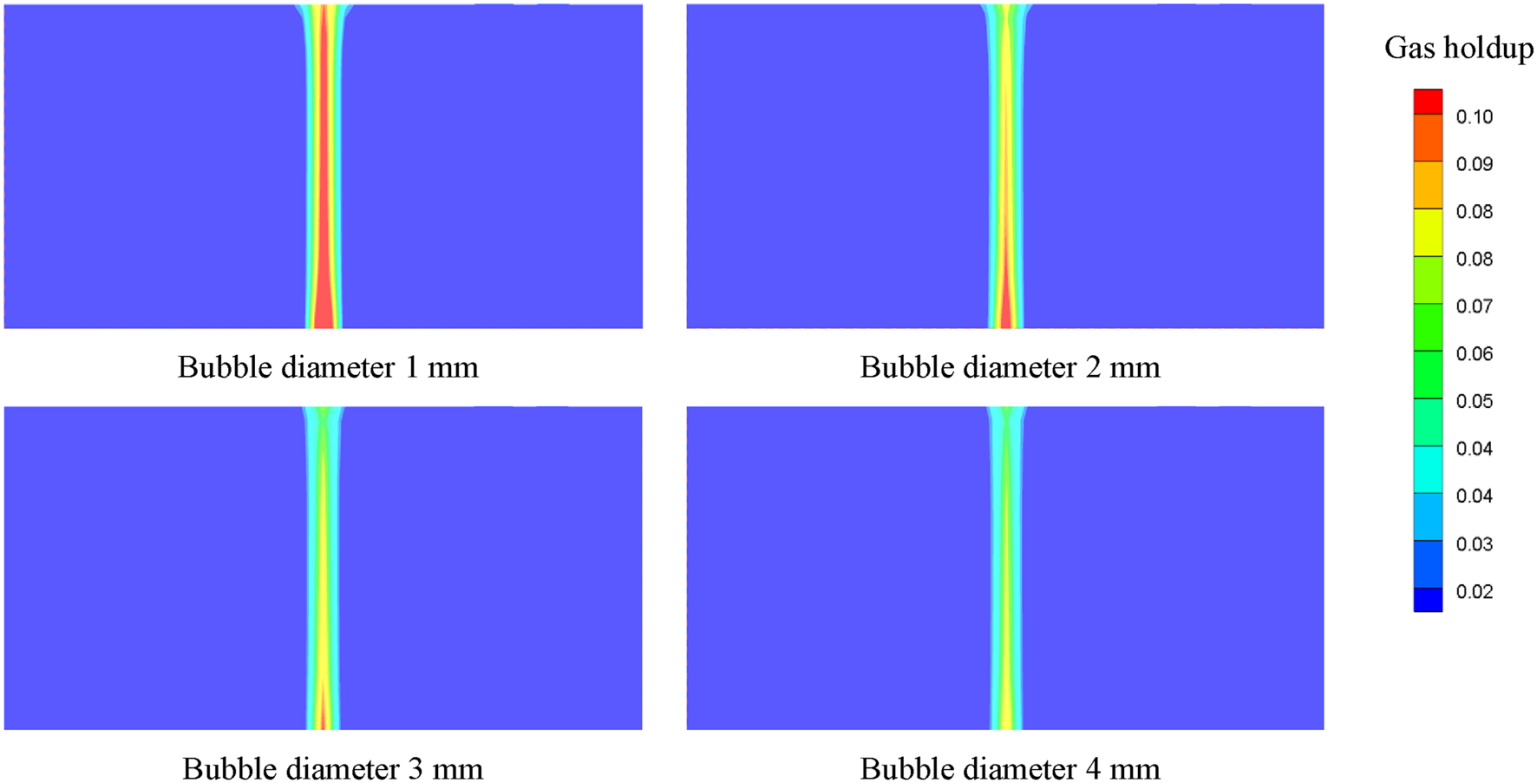
Gas holdup distributions at different bubble diameters.

The study of gas holdup distribution in aviation fuel during the scrubbing process can be helpful to understand the basic law of fluid flow^17^, and the gas–liquid contact area is closely related to the gas holdup, which affects the mass transfer rate of oxygen and nitrogen at the gas–liquid interface. Figure 7 shows the contours of the gas holdup distribution at different bubble diameters, and it can be seen that the gas holdup decreases as the bubble diameter increases. The average gas holdup in fuel is 1.352%, 1.315%, 1.283% and 1.234% separately at bubble diameters varying from 1 to 4 mm, and the maximum change is 8.73%.

**Figure 8:**
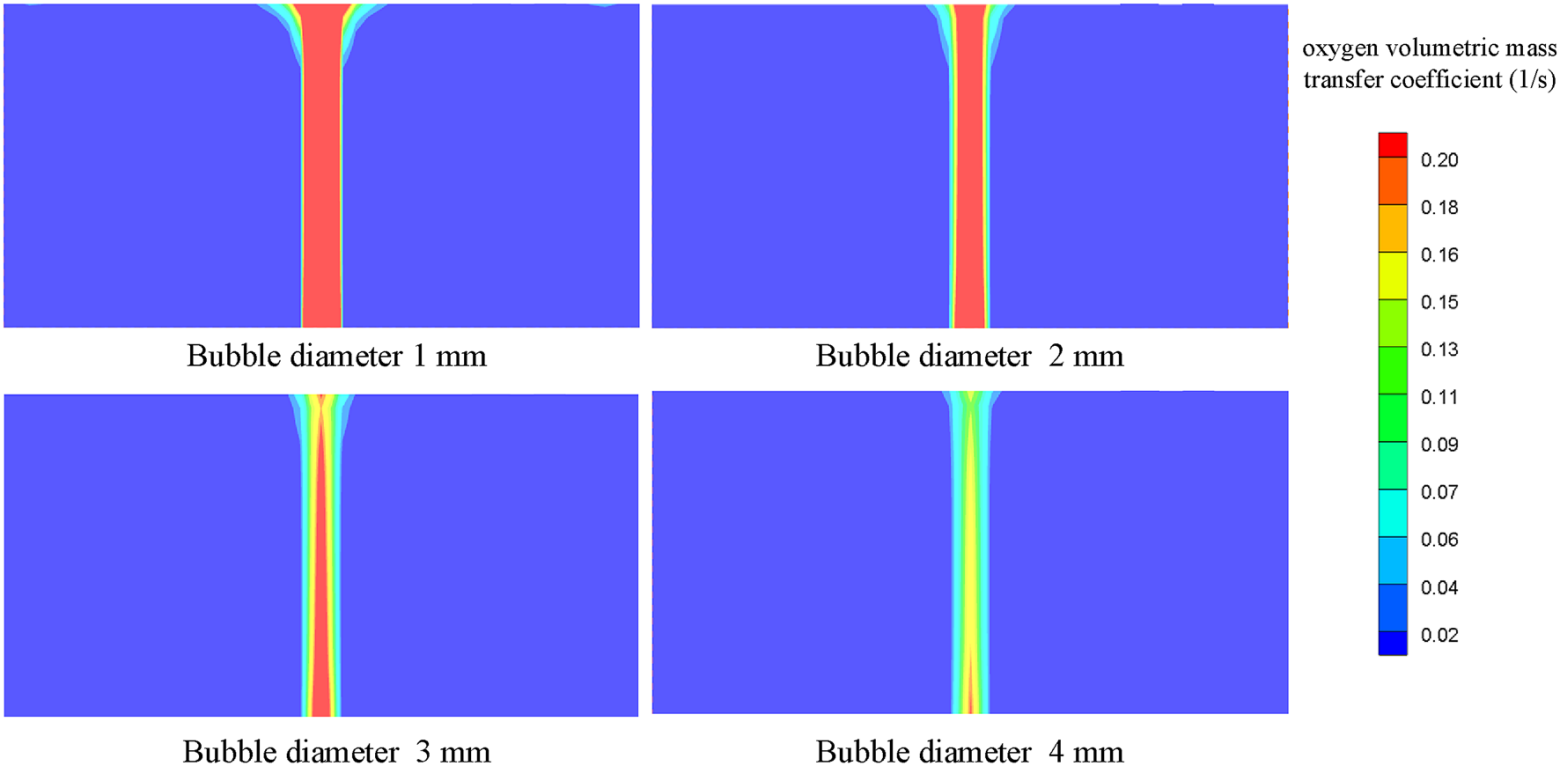
Oxygen volumetric mass transfer coefficient at different bubble diameters.

The oxygen volumetric mass transfer coefficient is defined as the product of the oxygen mass transfer coefficient and gas–liquid contact area that represent the performance of the scrubbing system. Figure 8 presents the variation of the oxygen volumetric mass transfer coefficient with bubble diameter. It is obvious that the oxygen volumetric mass transfer coefficient decreases with increasing bubble diameter. The average oxygen volumetric mass transfer coefficient is 0.221 1/s, 0.117 1/s, 0.080 1/s and 0.041 1/s separately at bubble diameters varying from 1 to 4 mm, and the maximum change is 81.45%.

### Influence of NEA superficial velocity

**Figure 9:**
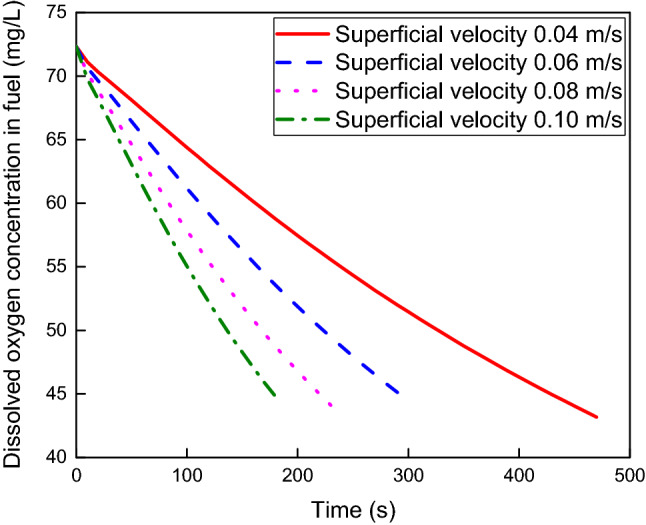
Dissolved oxygen concentration versus scrubbing time at various NEA superficial velocities.

The gas–liquid mass transfer in aviation fuel scrubbing is simulated with different NEA superficial velocities of 0.04 m/s, 0.06 m/s, 0.08 m/s and 0.10 m/s at an NEA bubble diameter of 2 mm and fuel load of 80%. Figure 9 shows the dissolved oxygen concentration versus scrubbing time. The dissolved oxygen concentration decreases with increasing scrubbing time, and the higher the NEA superficial velocity is, the lower the dissolved oxygen concentration. The maximum difference in dissolved oxygen concentration at the same scrubbing time can reach 23.78% at the four superficial velocities. The rate of descent of dissolved oxygen concentration rises as the NEA superficial velocity increases. The average rates of descent of dissolved oxygen concentration are 0.062 mg/s, 0.093 mg/s, 0.123 mg/s and 0.153 mg/s when the superficial velocity varies from 0.04 m/s to 0.10 m/s, and the maximum change is 146.77%.

**Figure 10:**
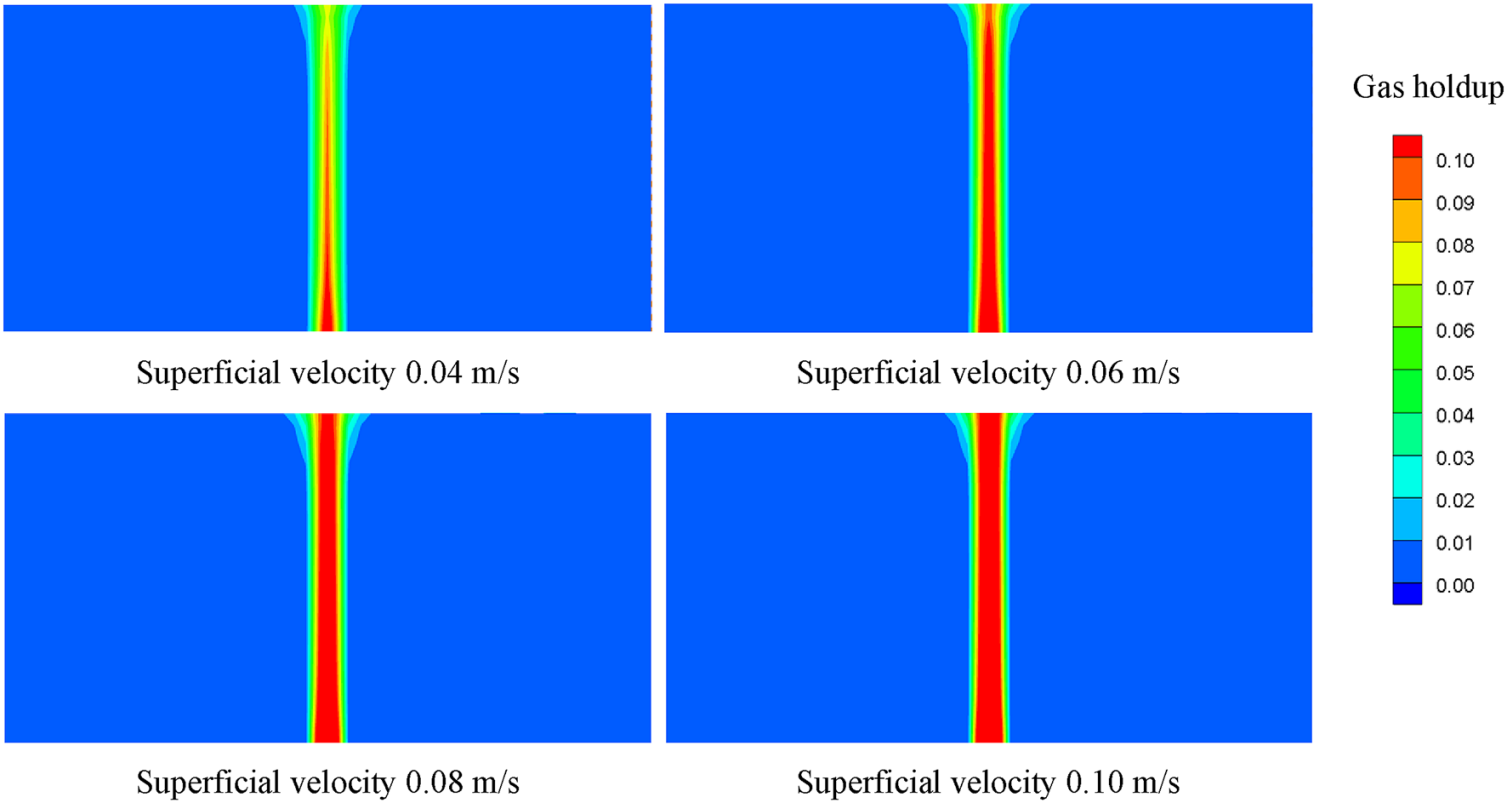
Gas holdup distributions at different superficial velocities.

**Figure 11:**
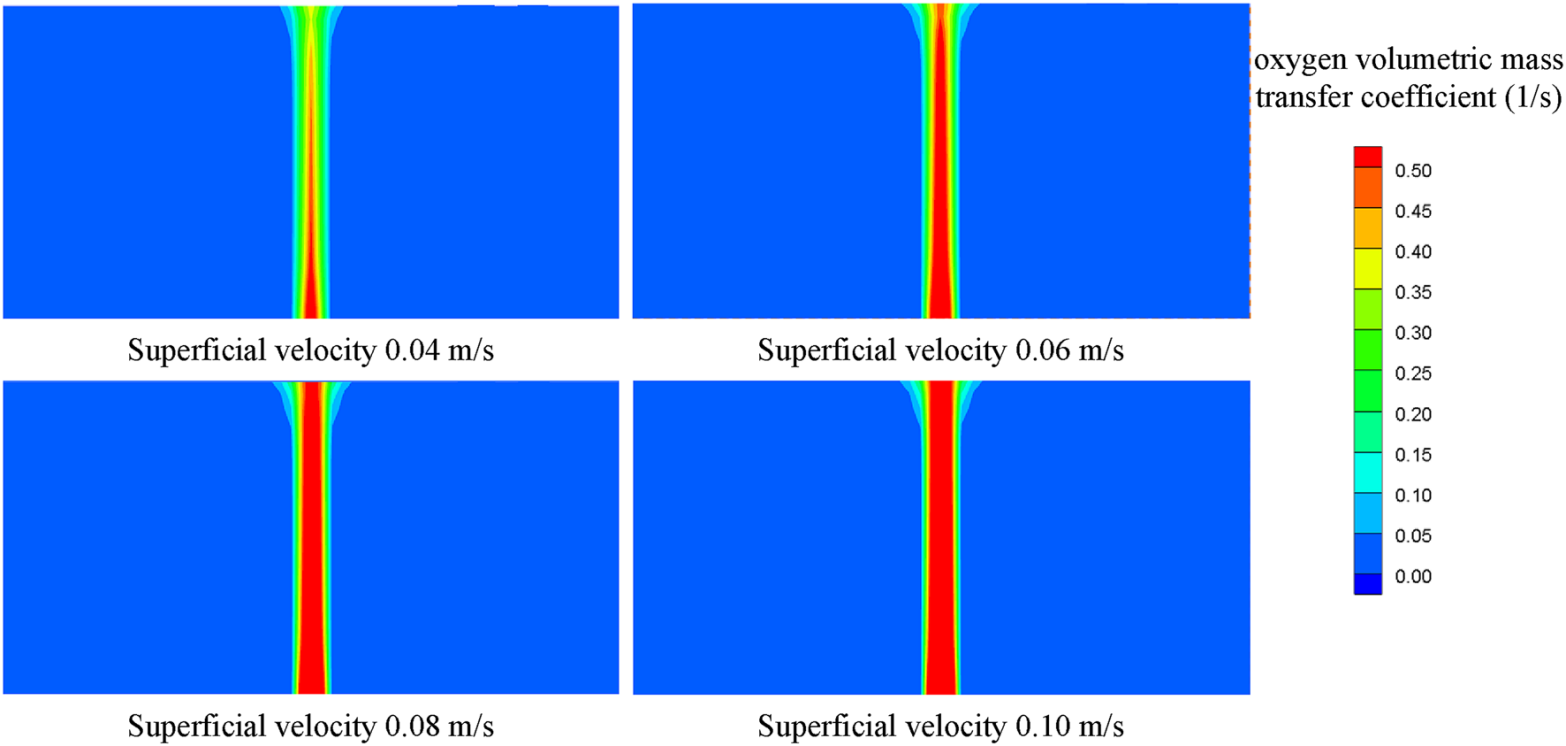
Oxygen volumetric mass transfer coefficients at different superficial velocities.

Figure 10 shows the contours of the gas holdup distribution at different superficial velocities, and the gas holdup increases with increasing superficial velocity. The average gas holdup in fuel is 1.076%, 1.315%, 1.512% and 1.906% at superficial velocities varying from 0.04 m/s to 0.10 m/s, and the maximum change is 77.14%. Figure 11 shows the variation of the oxygen volumetric mass transfer coefficient with superficial velocity. The oxygen volumetric mass transfer coefficient increases with increasing superficial velocity because the increase in superficial velocity results in an increase in the oxygen mass transfer coefficient and an increase in the gas–liquid contact area. The average oxygen volumetric mass transfer coefficient is 0.065 1/s, 0.117 1/s, 0.147 1/s and 0.179 1/s at superficial velocities varying from 0.04 m/s to 0.10 m/s, and the maximum change is 175.38%.

### Influence of fuel load

**Figure 12:**
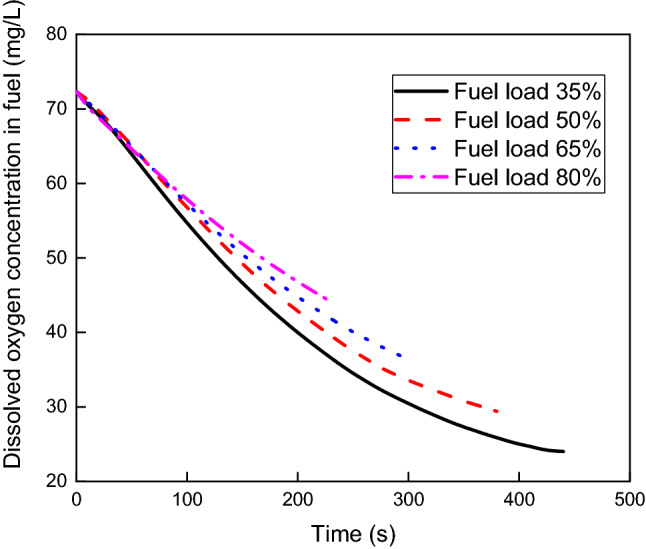
Dissolved oxygen concentration versus scrubbing time at various fuel loads.

Figure 12 shows the dissolved oxygen concentration versus scrubbing time at different fuel loads of 35%, 50%, 65% and 80% with an NEA bubble diameter of 2 mm and NEA superficial velocity of 0.08 m/s. The dissolved oxygen concentration also decreases with increasing scrubbing time, and the higher the fuel load is, the higher the dissolved oxygen concentration. The maximum difference in dissolved oxygen concentration at the same scrubbing time can reach 20.9%. The rate of descent of dissolved oxygen concentration decreases as the fuel load increases. The rates of descent of dissolved oxygen concentration are 0.156 mg/s, 0.143 mg/s, 0.133 mg/s and 0.123 mg/s when the fuel load varies from 35 to 80%, and the maximum change is 21.15%.

**Figure 13:**
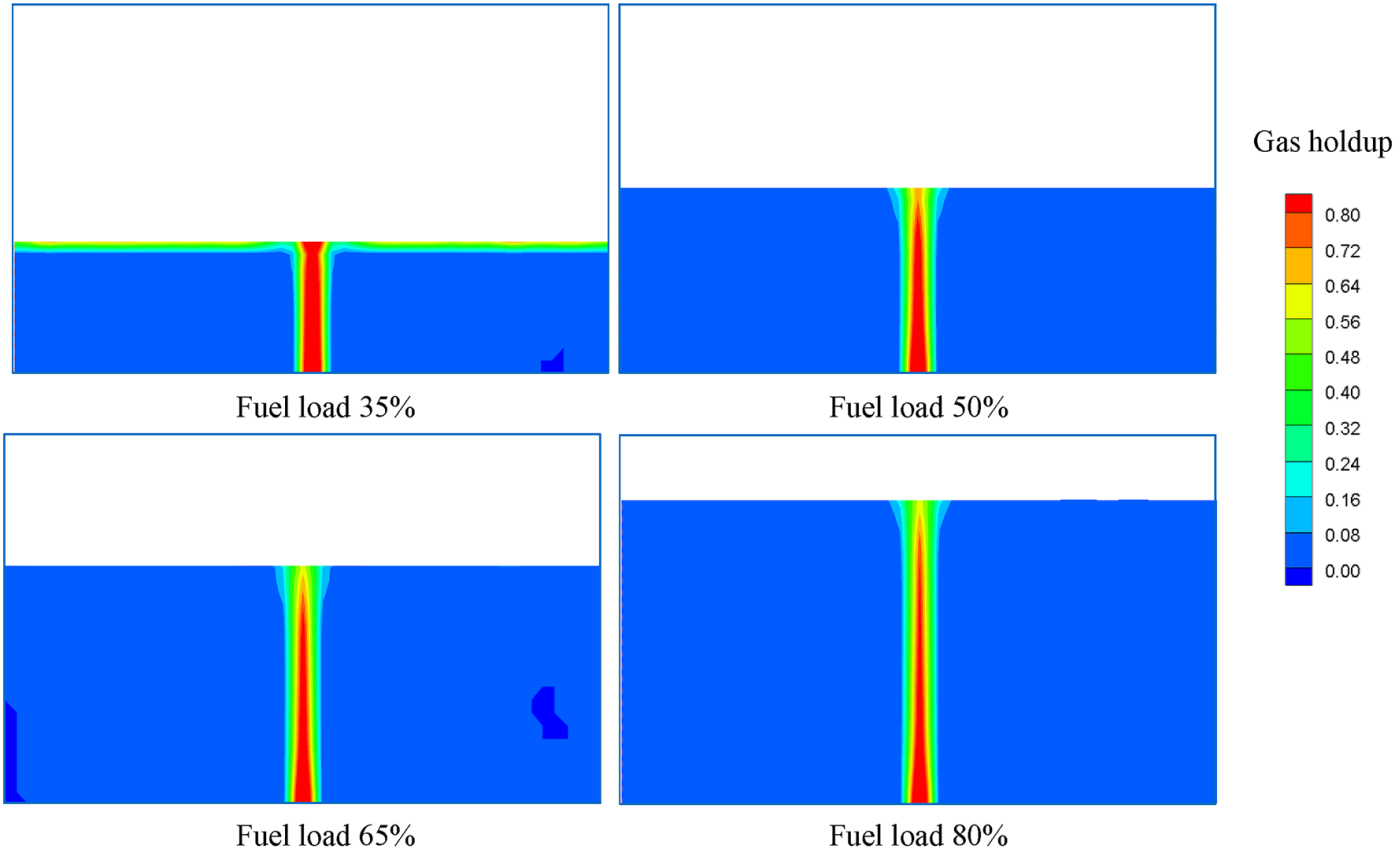
Gas holdup distributions at different fuel loads.

**Figure 14:**
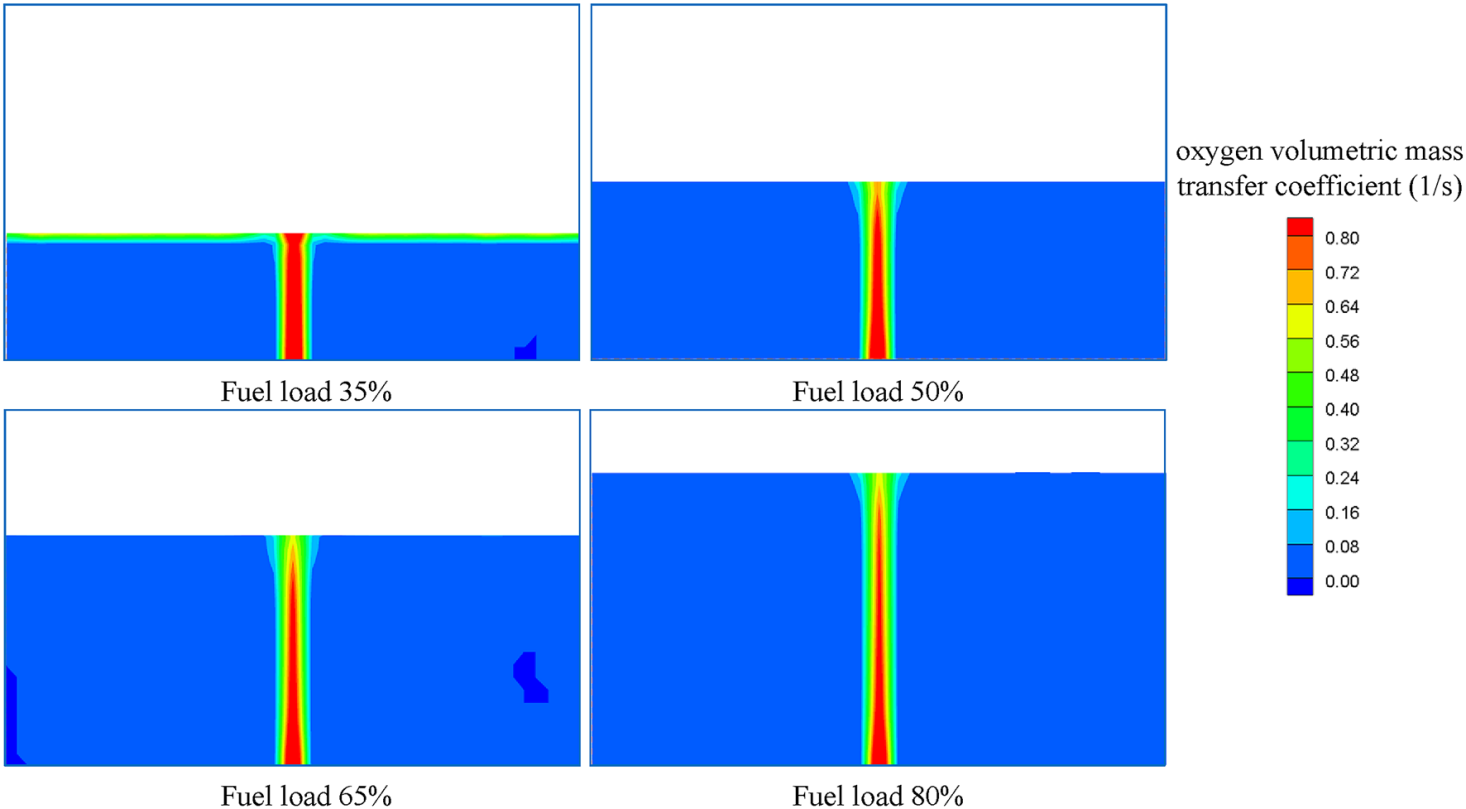
Oxygen volumetric mass transfer coefficients at different fuel loads.

The contours of the gas holdup distribution at different fuel loads are shown in Fig. 13. It is obvious that the gas holdup decreases with increasing fuel load. The average gas holdup in fuel is 2.719%, 2.387%, 1.753% and 1.372% at fuel loads varying from 35 to 80%, and the maximum change is 49.54%. Figure 14 presents the variation of the oxygen volumetric mass transfer coefficient with fuel load. The oxygen volumetric mass transfer coefficient decreases with increasing fuel load because the oxygen mass transfer coefficient and gas–liquid contact area both decrease. The average oxygen volumetric mass transfer coefficient is 0.267 1/s, 0.215 1/s, 0.186 1/s and 0.148 1/s separately as the fuel load varies from 35 to 80%, and the maximum change is 44.57%.

## Conclusions

The gas–liquid mass transfer characteristics of aviation fuel scrubbing in an aircraft fuel tank are simulated by the CFD method based on two fluid models. The correctness of the CFD method is verified by experiments with a deviation of 6.67%, and it is regarded that the mathematical model can be used to predict the behaviors of oxygen and nitrogen mass transfer between aviation fuel and NEA bubbles. The effects of the NEA bubble diameter, NEA superficial velocity and fuel load on the gas–liquid mass transfer characteristics are simulated and discussed. The dissolved oxygen concentration gradually decreases during aviation fuel scrubbing. The rate of descent of dissolved oxygen concentration, gas holdup and oxygen volumetric mass transfer coefficient all decrease as the bubble diameter and fuel load increase. In contrast, these values increase with increasing NEA superficial velocity.

## Ethical approval

The research for this article do not include human or animal subjects.

## Data Availability

All data generated or analysed during this study are included in this published article.

## List of symbols

*a*: Gas–liquid interface area (m^2^/m^3^)
*C*_D_: Drag coefficient
*C*_L_: Lift coefficient
*C*_VM_: Virtual mass coefficient
*c*_N_: Dissolved nitrogen concentrations in aviation fuel (kg/m^3^)
*c*_O_: Dissolved oxygen concentrations in aviation fuel (kg/m^3^)
*c*_s,N_: Saturation concentrations of dissolved nitrogen in the liquid phase corresponding to instantaneous partial pressure of gas N_2_ (kg/m^3^)
*c*_s,O_: Saturation concentrations of dissolved oxygen in the liquid phase corresponding to instantaneous partial pressure of gas O_2_ (kg/m^3^)
$c^{\prime}_{{\text{i}}} u^{\prime}_{{\text{i}}}$: Turbulent diffusion of the concentration (kg/(m^2^ s)
*D*_N_: Nitrogen mass diffusion coefficients in aviation (m^2^/s)
*D*_O_: Oxygen mass diffusion coefficients in aviation (m^2^/s)
*d*_B_: Bubble diameter (m)
*d*_bq_: Equivalent diameter of NEA bubble q (m)
*d*_bs_: Sauter diameter of the NEA bubble (m)
*E*o: Eotvos number
**g**: Gravitational acceleration (m/s^2^)
*h*_bq_: Major axis of the ellipsoidal bubble (m)
**J**_i_: Mass flux resulting from molecular diffusion (kg/(m^2^ s))
*K*_N_: Mass transfer coefficients of N_2_ in aviation fuel (m/s)
*K*_O_: Mass transfer coefficients of O_2_ in aviation fuel (m/s)
*k*: Turbulence kinetic energy (m^2^/s^2^)
*L*: Ostwald coefficient
*l*_bq_: Minor axis of the ellipsoidal bubble (m)
*n*_q_: Number of bubbles having an equivalent diameter *d*_bq_
*P*_k_: Turbulence product resulting from shear (m^2^/s^2^)
*p*: Pressure (Pa)
**R**: Interfacial force (N/m)
*R*e: Reynold number
*S*_i_: Interfacial mass transfer source (kg/m^3^)
*S*_N_: Interfacial mass transfer of N_2_ (kg/(m^3^ s))
*S*_O_: Interfacial mass transfer of O_2_ (kg/(m^3^ s))
*T*: Temperature (K)
*t*: Time (s)
**u**: Velocity (m/s)
*u*_r_: Relative velocity between the NEA bubble and liquid (m/s)
*α*: Volume fraction
*ρ*: Density (kg/m^3^)
*τ*: Stress tensors
*μ*: Dynamic viscosity (Pa s)
*μ*_t_: Turbulence dynamic viscosity (Pa s)
*ω*: Surface tension coefficient
*ε*: Dissipation rate of turbulence kinetic energy

## Superscript

DF: Drag force
LF: Lift force
VMF: Virtual mass force

## Subscript

i, j: The phase index
l: Liquid phase
g: Gas phase

## References

[1] Feng SY, Peng XT, Chen C (2020). Effect of air supplementation on the performance of an onboard catalytic inerting system. Aerosp. Sci. Technol..

[2] Keim M, Kallo J, Friedrich KA (2013). Multifunctional fuel cell system in an aircraft environment: An investigation focusing on fuel tank inerting and water generation. Aerosp. Sci. Technol..

[3] Pei Y, Shi B (2016). Method for analyzing the effect of projectile impact on aircraft fuel tank inerting for survivability design. Proc. Inst. Mech. Eng. Part G J. Aerosp. Eng..

[4] Cavage W, Bowman T. Modeling In-Flight Inert Gas Distribution in a 747 Center Wing Fuel Tank: 35th AIAA Fluid Dynamics Conference and Exhibit Toronto 2005; Ontario Canada.

[5] Li CY, Feng SY, Chen C (2019). Performance analysis of aircraft fuel tank inerting system with turbocharger. Proc. Inst. Mech. Eng. Part G J. Aerosp. Eng..

[6] Renouard-Vallet GNL, Saballus M, Schmithals G (2010). Improving the environmental impact of civil aircraft by fuel cell technology: concepts and technological progress. Energy Environ. Sci..

[7] Feng SY, Li CY, Peng XT (2020). Oxygen concentration variation in ullage influenced by dissolved oxygen evolution. Chin. J. Aeronaut..

[8] Shao L, Liu WH, Li CY (2018). Experimental comparison between aircraft fuel tank inerting processes using NEA and MIG. Chin. J. Aeronaut..

[9] Cavage WM. The effect of fuel on an Inert ullage in a commercial transport airplane fuel tank Washington. Washington, D.C.: Federal Aviation Administration 2005; Report No.: DOT/FAA/AR-05/25.

[10] Yang C, Mao Z (2005). Numerical simulation of interphase mass transfer with the level set approach. Chem. Eng. Sci..

[11] Bordel S, Mato R, Villaverde S (2006). Modeling of the evolution with length of bubble size distributions in bubble columns. Chem. Eng. Sci..

[12] Gillot S, Capela S, Heduit A (2000). Effect of horizontal flow on oxygen transfer in clean water and in clean water with surfactants. Water Res..

[13] Kulkarni AA (2007). Mass transfer in bubble column reactors: Effect of bubble size distribution. Ind. Eng. Chem. Res..

[14] Buwa VV, Ranade VV (2002). Dynamics of gas-liquid flow in a rectangular bubble column experiments and singlemulti-group CFD simulations. Chem. Eng. Sci..

[15] Trivedi R, Renganathan T, Krishnaiah K (2018). Hydrodynamics of countercurrent bubble column: Experiments and predictions. Chem. Eng. J..

[16] McClure DD, Kavanagh JM, Fletcher DF (2015). Oxygen transfer in bubble columns at industrially relevant superficial velocities: Experimental work and CFD modelling. Chem. Eng. J..

[17] Terashima M, So M, Goel R (2016). Determination of diffuser bubble size in computational fluid dynamics models to predict oxygen transfer in spiral roll aeration tanks. J. Water Process Eng..

[18] Fayolle Y, Cockx A, Gillot S (2007). Oxygen transfer prediction in aeration tanks using CFD. Chem. Eng. Sci..

[19] Wen T, Lu L, Dong C (2018). Investigation on the regeneration performance of liquid desiccant by adding surfactant PVP-K30. Int. J. Heat Mass Transf..

[20] Talvy S, Cockx A, Line A (2007). Modeling hydrodynamics of gas–liquid airlift reactor. AIChE J..

[21] Gresch M, Armbruster M, Braun D (2011). Effects of aeration patterns on the flow field in wastewater aeration tanks. Water Res..

[22] Saleh SN, Mohammed AA, Al-Jubory FK (2018). CFD assesment of uniform bubbly flow in a bubble column. J. Petrol. Sci. Eng..

[23] Mellin P, Zhang QL, Kantarelis E (2013). An Euler-Euler approach to modeling biomass fast pyrolysis in fluidized-bed reactors: Focusing on the gas phase. Appl. Therm. Eng..

[24] Pu WH, Yang N, Yue C (2019). Simulation on direct contact heat transfer in gas-molten salt bubble column for high temperature solar thermal storage. Int. Commun. Heat Mass Transfer.

[25] Liu Y, Zhang H, Wang S (2008). Prediction of pressure gradient and holdup in small Eötvös number Liquid–Liquid segregated flow. Chin. J. Chem. Eng..

[26] Montes-Atenas G, Seguel F, Valencia A (2016). Predicting bubble size and bubble rate data in water and in froth flotation-like slurry from computational fluid dynamics (CFD) by applying deep neural networks (DNN). Int. Commun. Heat Mass Transfer.

[27] Tomiyama A, Tamai H, Zun I (2002). Transverse migration of single bubbles in simple shear flows. Chem. Eng. Sci..

[28] Mougin G, Magnaudet J (2002). The generalized Kirchhoff equations and their application to the interaction between a rigid body and an arbitrary time-dependent viscous flow. Int. J. Multiph. Flow.

[29] Bridgeman J, Jefferson B, Parsons SA (2009). Computational fluid dynamics modelling of flocculation in water treatment: A review. Eng. Appl. Comput. Fluid Mech..

[30] Karpinska AM, Bridgeman J (2016). CFD-aided modelling of activated sludge systems: A critical review. Water Res..

[31] Xu N, Fan L, Pang H (2010). Feasibility study and CFD-aided design for a new type oxidation ditch based on airlift circulation. Can. J. Chem. Eng..

[32] Sun W, Zhu C, Fu T (2019). 3D simulation of interaction and drag coefficient of bubbles continuously rising with equilateral triangle arrangement in shear-thinning fluids. Int. J. Multiph. Flow.

[33] Shi W, Yang N, Yang X (2017). A kinetic inlet model for CFD simulation of large-scale bubble columns. Chem. Eng. Sci..

[34] Committee ASTM. Standard Test Method for Estimation of Solubility of Gases in Petroleum Liquids. Annual book of ASTM standards. West Conshohocken: ASTM International; 2002.

[35] Higbie R (1935). The rate of adsorption of a pure gas into a still liquid during short period of exposure. Trans. Am. Inst. Chem. Eng..

[36] Behzadfar E, Hatzikiriakos SG (2014). Diffusivity of CO_2_ in Bitumen: Pressure–decay measurements coupled with rheometry. Energy Fuels.

[37] Avgoustiniatos ES, Dionne KE, Wilson DF (2007). Measurements of the effective diffusion coefficient of oxygen in pancreatic islets. Ind. Eng. Chem. Res..

[38] Li CY, Feng SY, Shao L (2019). Experimental study of the solubility and diffusivity of CO2and O2 in RP-3 jet fuel. Aircr. Eng. Aerosp. Technol..

[39] Li CY, Liu WH, Peng XT (2019). Measurement of mass diffusion coefficients of O2 in aviation fuel through digital holographic interferometry. Chin. J. Aeronaut..

[40] Shao L, Feng S, Li C (2019). Effect of scrubbing efficiency on fuel scrubbing inerting for aircraft fuel tanks. Aircr. Eng. Aerosp. Technol..

